# Supplementary material for: Resolving the full spectrum of human genome variation using Linked-Reads
Source: Genome Res. 2019 Apr;29(4):635–45. doi: 10.1101/gr.234443.118 (PMC6442396; doi:10.1101/gr.234443.118)
Supplement: Supplemental Material [file supp_gr.234443.118_Supplemental_longranger-2.2.zip › longranger-2.2.tar.bz2!/lr2.2-public/lib/stan/cmdstan/stan/doc/api/html/functions_type.html]

Stan: Class Members - Typedefs


|  |  |
| --- | --- |
|  | Stan  2.5.0  probability, sampling & optimization |


- Main Page
- Related Pages
- Namespaces
- Classes
- Files

- Class List
- Class Index
- Class Hierarchy
- Class Members

- All
- Functions
- Variables
- Typedefs
- Enumerator
- Related Functions

- a
- b
- e
- h
- i
- l
- m
- n
- p
- r
- s
- t
- u
- v


All Classes Namespaces Files Functions Variables Typedefs Enumerations Enumerator Friends Macros Pages

### - a -

- array\_vec\_t
  : stan::io::writer< T >

### - b -

- BFGSBase
  : stan::optimization::BFGSLineSearch< M, QNUpdateType, Scalar, DimAtCompile >

### - e -

- expression\_t
  : stan::gm::expression

### - h -

- HessianT
  : stan::optimization::BFGSMinimizer< FunctorType, QNUpdateType, Scalar, DimAtCompile >
  , stan::optimization::BFGSUpdate\_HInv< Scalar, DimAtCompile >
  , stan::optimization::LBFGSUpdate< Scalar, DimAtCompile >

### - i -

- idx\_t
  : stan::optimization::BFGSLineSearch< M, QNUpdateType, Scalar, DimAtCompile >

### - l -

- LhsScalar
  : Eigen::internal::general\_matrix\_matrix\_product< Index, stan::agrad::var, LhsStorageOrder, ConjugateLhs, stan::agrad::var, RhsStorageOrder, ConjugateRhs, ColMajor >
  , Eigen::internal::general\_matrix\_vector\_product< Index, stan::agrad::var, ColMajor, ConjugateLhs, stan::agrad::var, ConjugateRhs >
  , Eigen::internal::general\_matrix\_vector\_product< Index, stan::agrad::var, RowMajor, ConjugateLhs, stan::agrad::var, ConjugateRhs >

### - m -

- map\_matrix\_t
  : stan::io::reader< T >
- map\_row\_vector\_t
  : stan::io::reader< T >
- map\_vector\_t
  : stan::io::reader< T >
- matrix\_t
  : stan::io::reader< T >
  , stan::io::writer< T >
  , stan::VectorViewMvt< T, is\_array, throw\_if\_accessed >
  , stan::VectorViewMvt< const T, is\_array, throw\_if\_accessed >

### - n -

- Nested
  : Eigen::NumTraits< stan::agrad::var >
- NonInteger
  : Eigen::NumTraits< stan::agrad::var >

### - p -

- printable\_t
  : stan::gm::printable

### - r -

- range\_t
  : stan::gm::variable\_map
- Real
  : Eigen::NumTraits< stan::agrad::var >
- ResScalar
  : Eigen::internal::general\_matrix\_matrix\_product< Index, stan::agrad::var, LhsStorageOrder, ConjugateLhs, stan::agrad::var, RhsStorageOrder, ConjugateRhs, ColMajor >
  , Eigen::internal::general\_matrix\_vector\_product< Index, stan::agrad::var, ColMajor, ConjugateLhs, stan::agrad::var, ConjugateRhs >
  , Eigen::internal::general\_matrix\_vector\_product< Index, stan::agrad::var, RowMajor, ConjugateLhs, stan::agrad::var, ConjugateRhs >
- result\_type
  : stan::gm::visgen
- ReturnType
  : Eigen::internal::scalar\_product\_traits< double, stan::agrad::var >
  , Eigen::internal::scalar\_product\_traits< stan::agrad::var, double >
- RhsScalar
  : Eigen::internal::general\_matrix\_matrix\_product< Index, stan::agrad::var, LhsStorageOrder, ConjugateLhs, stan::agrad::var, RhsStorageOrder, ConjugateRhs, ColMajor >
  , Eigen::internal::general\_matrix\_vector\_product< Index, stan::agrad::var, ColMajor, ConjugateLhs, stan::agrad::var, ConjugateRhs >
  , Eigen::internal::general\_matrix\_vector\_product< Index, stan::agrad::var, RowMajor, ConjugateLhs, stan::agrad::var, ConjugateRhs >
- row\_vector\_t
  : stan::io::reader< T >
  , stan::io::writer< T >

### - s -

- Scalar
  : stan::agrad::var
- scalar\_t
  : stan::VectorView< T, is\_array, throw\_if\_accessed >
  , stan::VectorView< const T, is\_array, throw\_if\_accessed >
- size\_type
  : stan::math::LDLT\_factor< stan::agrad::var, R, C >
  , stan::math::LDLT\_factor< T, R, C >
- statement\_t
  : stan::gm::statement

### - t -

- type
  : add\_var::result< T1, T2, T3, T4, T5, T6 >
  , copy\_square\_cholesky\_dimension\_if\_necessary::result< T1 >
  , empty\_range::result< T1 >
  , set\_double\_range\_lower::result< T1, T2, T3, T4 >
  , set\_double\_range\_upper::result< T1, T2, T3, T4 >
  , set\_int\_range\_lower::result< T1, T2, T3, T4 >
  , set\_int\_range\_upper::result< T1, T2, T3, T4 >
  , stan::gm::add\_conditional\_body::result< T1, T2 >
  , stan::gm::add\_conditional\_condition::result< T1, T2, T3, T4 >
  , stan::gm::add\_expression\_dimss::result< T1, T2, T3, T4 >
  , stan::gm::add\_fun\_var::result< T1, T2, T3, T4 >
  , stan::gm::add\_function\_signature::result< T1, T2, T3, T4, T5 >
  , stan::gm::add\_loop\_identifier::result< T1, T2, T3, T4 >
  , stan::gm::add\_while\_body::result< T1, T2 >
  , stan::gm::add\_while\_condition::result< T1, T2, T3 >
  , stan::gm::addition\_expr3::result< T1, T2, T3 >
  , stan::gm::binary\_op\_expr::result< T1, T2, T3, T4, T5 >
  , stan::gm::division\_expr::result< T1, T2, T3 >
  , stan::gm::elt\_division\_expr::result< T1, T2, T3 >
  , stan::gm::elt\_multiplication\_expr::result< T1, T2, T3 >
  , stan::gm::exponentiation\_expr::result< T1, T2, T3, T4, T5 >
  , stan::gm::expression\_as\_statement::result< T1, T2, T3 >
  , stan::gm::left\_division\_expr::result< T1, T2, T3 >
  , stan::gm::logical\_negate\_expr::result< T1, T2, T3 >
  , stan::gm::modulus\_expr::result< T1, T2, T3, T4 >
  , stan::gm::multiplication\_expr::result< T1, T2, T3 >
  , stan::gm::negate\_expr::result< T1, T2, T3, T4 >
  , stan::gm::remove\_loop\_identifier::result< T1, T2 >
  , stan::gm::scope\_lp::result< T1 >
  , stan::gm::set\_allows\_sampling\_origin::result< T1, T2, T3 >
  , stan::gm::set\_fun\_type2::result< T1, T2 >
  , stan::gm::set\_fun\_type3::result< T1, T2 >
  , stan::gm::set\_fun\_type::result< T1, T2 >
  , stan::gm::set\_fun\_type\_named::result< T1, T2, T3, T4, T5 >
  , stan::gm::set\_var\_type::result< T1, T2, T3, T4 >
  , stan::gm::set\_void\_function::result< T1, T2, T3, T4 >
  , stan::gm::subtraction\_expr3::result< T1, T2, T3 >
  , stan::gm::transpose\_expr::result< T1, T2 >
  , stan::gm::unscope\_locals::result< T1, T2 >
  , stan::gm::unscope\_variables::result< T1, T2 >
  , stan::gm::validate\_allow\_sample::result< T1, T2, T3 >
  , stan::gm::validate\_assignment::result< T1, T2, T3, T4 >
  , stan::gm::validate\_declarations::result< T1, T2, T3, T4 >
  , stan::gm::validate\_expr\_type3::result< T1, T2 >
  , stan::gm::validate\_int\_expr2::result< T1, T2, T3 >
  , stan::gm::validate\_int\_expr3::result< T1, T2 >
  , stan::gm::validate\_integrate\_ode::result< T1, T2, T3, T4 >
  , stan::gm::validate\_non\_void\_arg\_function::result< T1, T2, T3 >
  , stan::gm::validate\_non\_void\_expression::result< T1, T2, T3 >
  , stan::gm::validate\_return\_allowed::result< T1, T2, T3 >
  , stan::gm::validate\_return\_type::result< T1, T2, T3 >
  , stan::gm::validate\_sample::result< T1, T2, T3 >
  , stan::gm::validate\_void\_return\_allowed::result< T1, T2, T3 >
  , stan::is\_vector< T >
  , stan::is\_vector< const T >
  , stan::is\_vector< Eigen::Block< T > >
  , stan::is\_vector< Eigen::Matrix< T, 1, Eigen::Dynamic > >
  , stan::is\_vector< Eigen::Matrix< T, Eigen::Dynamic, 1 > >
  , stan::is\_vector< std::vector< T > >
  , stan::math::common\_type< T1, T2 >
  , stan::math::common\_type< Eigen::Matrix< T1, R, C >, Eigen::Matrix< T2, R, C > >
  , stan::math::common\_type< std::vector< T1 >, std::vector< T2 > >
  , stan::math::index\_type< const T >
  , stan::math::index\_type< Eigen::Matrix< T, R, C > >
  , stan::math::index\_type< std::vector< T > >
  , stan::math::pass\_type< T >
  , stan::math::pass\_type< double >
  , stan::math::pass\_type< int >
  , stan::math::promote\_scalar\_type< T, S >
  , stan::math::promote\_scalar\_type< T, Eigen::Matrix< S, 1, Eigen::Dynamic > >
  , stan::math::promote\_scalar\_type< T, Eigen::Matrix< S, Eigen::Dynamic, 1 > >
  , stan::math::promote\_scalar\_type< T, Eigen::Matrix< S, Eigen::Dynamic, Eigen::Dynamic > >
  , stan::math::promote\_scalar\_type< T, std::vector< S > >
  , stan::math::store\_type< T >
  , stan::math::store\_type< double >
  , stan::math::store\_type< int >
  , stan::math::value\_type< const T >
  , stan::math::value\_type< Eigen::Matrix< T, R, C > >
  , stan::math::value\_type< std::vector< T > >
  , stan::return\_type< T1, T2, T3, T4, T5, T6 >
  , stan::scalar\_type< T >
  , stan::scalar\_type< Eigen::Matrix< T, Eigen::Dynamic, Eigen::Dynamic > >
  , stan::scalar\_type< T \* >
  , stan::scalar\_type\_pre< T >
  , validate\_decl\_constraints::result< T1, T2, T3, T4 >
  , validate\_double\_expr::result< T1, T2 >
  , validate\_identifier::result< T1, T2 >
  , validate\_int\_data\_expr::result< T1, T2, T3, T4, T5 >
  , validate\_int\_expr::result< T1, T2, T3 >

### - u -

- UpdateT
  : stan::optimization::LBFGSUpdate< Scalar, DimAtCompile >

### - v -

- value\_type
  : stan::agrad::fvar< T >
  , stan::math::LDLT\_factor< stan::agrad::var, R, C >
  , stan::math::LDLT\_factor< T, R, C >
- var\_context\_t
  : stan::common::var\_context\_factory< VARCON >
- var\_decl\_t
  : stan::gm::var\_decl
- vector\_t
  : stan::io::reader< T >
  , stan::io::writer< T >
  , stan::optimization::BFGSLineSearch< M, QNUpdateType, Scalar, DimAtCompile >
- VectorT
  : stan::optimization::BFGSMinimizer< FunctorType, QNUpdateType, Scalar, DimAtCompile >
  , stan::optimization::BFGSUpdate\_HInv< Scalar, DimAtCompile >
  , stan::optimization::LBFGSUpdate< Scalar, DimAtCompile >


---

[ Stan Home Page ]

*© 2011–2014,
Stan Development Team.*
